# Feasibility of a home-based interdisciplinary rehabilitation program for patients with Post-Intensive Care Syndrome: the REACH study

**DOI:** 10.1186/s13054-021-03709-z

**Published:** 2021-08-05

**Authors:** Mel E. Major, Daniela Dettling-Ihnenfeldt, Stephan P. J. Ramaekers, Raoul H. H. Engelbert, Marike van der Schaaf

**Affiliations:** 1 European School of Physiotherapy, Faculty of Health, Amsterdam University of Applied Sciences, Amsterdam, The Netherlands; 2 Faculty of Health, Center of Expertise Urban Vitality, Amsterdam University of Applied Sciences, Amsterdam, The Netherlands; 3 Department of Rehabilitation Medicine, Amsterdam UMC, University of Amsterdam, Amsterdam Movement Sciences, Meibergdreef 9, Amsterdam, The Netherlands

**Keywords:** Post-Intensive Care Syndrome, Critical illness, Rehabilitation, Physical therapy, Recovery, Feasibility, Pilot

## Abstract

**Background:**

Survivors of critical illness experience long-term functional challenges, which are complex, heterogeneous, and multifactorial in nature. Although the importance of rehabilitation interventions after intensive care unit (ICU) discharge is universally recognized, evidence on feasibility and effectiveness of home-based rehabilitation programs is scarce and ambiguous. This study investigates the feasibility of an interdisciplinary rehabilitation program designed for patients with Post-Intensive Care Syndrome (PICS) who are discharged home.

**Methods:**

A mixed method, non-randomized, prospective pilot feasibility study was performed with a 6-month follow-up, comparing the intervention (REACH) with usual care. REACH was provided by trained professionals and included a patient-centered, interdisciplinary approach starting directly after hospital discharge. Primary outcomes were patient safety, satisfaction, adherence, referral need and health care usage. Secondary outcomes, measured at 3 timepoints, were functional exercise capacity, self-perceived health status, health-related quality of life (HRQoL), return to work and psychotrauma. Risk of undernutrition was assessed at baseline.

**Results:**

43 patients with a median mechanical ventilation duration of 8 (IQR:10) days, were included in the study and 79.1% completed 6-month follow-up. 19 patients received the intervention, 23 received usual care. Groups were similar for gender distribution and ICU length of stay. No adverse events occurred. REACH participants showed higher satisfaction with treatment and reported more allied health professional visits, while the usual care group reported more visits to medical specialists. Qualitative analysis identified positive experiences among REACH-professionals related to providing state-of-the-art interventions and sharing knowledge and expertise within an interprofessional network. Similar recovery was seen between groups on all secondary outcomes, but neither group reached reference values for HRQoL at 6 months. Larger return to work rates were seen in the REACH group. Prevalence of undernutrition at hospital discharge was high in both groups (> 80%), warranting the need for careful tuning of physical therapy and nutritional interventions.

**Conclusions:**

This study shows that providing early, home-based rehabilitation interventions for patients with PICS-related symptoms is feasible and perceived positively by patients and professionals. When provided in an interdisciplinary collaborative network state of the art, person-centered interventions can be tailored to individual needs potentially increasing patient satisfaction, adherence, and efficacy.

Registered in the Dutch Trial register: NL7792: https://www.trialregister.nl/trial/7792, registered 7-06-2019.

**Supplementary Information:**

The online version contains supplementary material available at 10.1186/s13054-021-03709-z.

## Background

Whilst more patients survive critical illness because of improvements in medical care, a growing number of patients leaves the hospital needing rehabilitation interventions for multifactorial problems associated with long-term disability as part of the Post-Intensive Care Syndrome (PICS) [1–6]. The new or worsened impairments reported by survivors of critical illness manifest in considerable heterogeneity with regards to health domains (physical, psychological, cognitive), duration, and severity of activity limitations and participation restrictions in life situations [7–12].

Although a range of interventions within the intensive care unit (ICU) is employed targeting these long-term functional problems, such as early mobilization and the use of ICU diaries, a rehabilitation continuum or coordinated care pathway after ICU- and hospital discharge is lacking [13, 14]. The diversity of problems survivors might experience warrant the need for an interdisciplinary approach towards recovery to provide tailor-made, individualized interventions at the right time, in the right setting and by the right professional [12, 15–20].

The World Health Organization's (WHO) definition of rehabilitation describes the importance of providing interventions directed towards interaction within the individual's *environment*, to facilitate participation in meaningful activities [21]. To date, few studies investigated interventions for patients recovering from critical illness after home discharge, and reported poor attendance of outpatient exercise programs. Travel time and patients' lack of motivation were identified as reasons for non-attendance [22–24]. If primary care rehabilitation specialists such as physical therapists (PTs), occupational therapists (OTs) and dietitians (DTs) can provide early home-based interventions for patients with functional impairments related to PICS, this might increase adherence and satisfaction, decrease the chance of hospital readmissions, and cut healthcare costs [12, 23, 25–27]. Care provided within an interprofessional network has shown to increase professional expertise and improve the quality of care [28, 29].

While expert recommendations for home-based, PT-led interventions for survivors of critical illness have been published [26, 30, 31], feasibility of such interventions within the primary care setting is yet to be investigated. Therefore, the aim of this study was to investigate the feasibility of an interdisciplinary home-based intervention for patients with new or worsened impairments within one of the domains of PICS, initiated immediately after hospital discharge and targeting (physical) recovery and self-management in comparison to patients receiving usual care.

## Methods

### Study design

A mixed method, non-randomized, prospective pilot feasibility study was undertaken with a 6-month follow up and a total study duration of 22 months. The pilot feasibility study consisted of two arms, an intervention group (REACH, REhabilitation After Critical illness and Hospital discharge) and a usual care group. Group allocation was based on convenience sampling; participants received the intervention if they lived in an area covered by REACH-therapists, unless they preferred otherwise (i.e., their own therapist). Participants living outside of the REACH geographical area were allocated to the usual care group. In line with the pilot feasibility character of the study, no a priori sample size calculations were conducted [32].

### Setting

This study was part of ongoing research of the department of rehabilitation medicine at the Amsterdam University Medical Centers, location Academic Medical Center (AMC).

### Participants

Participants were recruited from 2 university and 5 general hospitals in the Amsterdam area, the Netherlands. Participants were eligible for inclusion if they had received mechanical ventilation (MV) of  ≥ 48 h in the ICU, had developed new or worsened impairments during or after the ICU-stay unrelated to the initial admission diagnosis [33], and were discharged home with an indication for physical therapy (PT). Indication for PT was determined according to the hospitals' protocols for referral, i.e., presence of any (or a combination) of the following: ICU-acquired weakness (MRC Sum Score < 48), limited walking ability (Functional Ambulation Categories (FAC) ≤ 4), problems with climbing stairs, decreased independence in activities of daily living (ADL), limited cardiopulmonary capacity during exertion (dyspnea, resting respiratory rate > 30, oxygen saturation < 95%, Borg CR10 scale > 4).

Exclusion criteria were presence of serious (preexisting) cognitive and/or psychiatric impairments hindering compliance to the physical tests and inadequate understanding of the Dutch or English language. Eligible patients were identified by ICU-PTs and after verbal permission was obtained, contacted by telephone by the primary investigator (MM) within 2 days after hospital discharge. Once oral consent was obtained, a home visit was planned.

### Intervention

The intervention, called the *REACH program*, was designed in an iterative, 8-month developmental process in a community of practice (CoP) of primary care PTs (*n* = 18), OTs (*n* = 3), DTs (*n* = 4), ICU-PTs (*n* = 8), researcher/clinicians (*n* = 6), a health coach (*n* = 1) and representatives from patient- and professional organizations (including general practitioners), prior to the start of the study. First, CoP members from different fields of expertise provided training on the presentation and potential interventions for the different facets of PICS. Next, the components of the REACH program were developed in co-creation. Lastly, the ‘positive health’ concept was integrated in the REACH program. This concept emphasizes support to the ‘ability to adapt and self-manage’ [34]. Professionals within the REACH-network received extensive training with regards to the application of this concept of health in their daily practice, allowing for individualized, tailor-made treatment programs. The intervention, which was initiated by the hospital PTs, constituted an elaborate written and telephonic handover from hospital PT to REACH-PT, a core outcome set (CoS) and a tailored exercise program. Regular (online) CoP meetings facilitated peer-to-peer learning and interdisciplinary collaboration.

The PT interventions started within one week after hospital discharge, initially provided in the home situation of the patient and continuing in the nearby PT practice as soon as their physical condition allowed. During the first intake and/or during the treatment period PTs performed a screening to detect functional problems within the field of OT and DT and referred patients when indicated. The Short Nutritional Assessment Questionnaire (SNAQ65+) was used to screen for (the risk of) undernutrition [35], in which case DTs were consulted, who performed further diagnostics. DT interventions were targeted towards optimization of protein intake, according to the Dutch guidelines for malnutrition: 1.2–1.5 g protein per kg bodyweight [36] in participants in which undernutrition and/or sarcopenia were identified. For OT, 4 screening questions were designed as advised by expert OTs within the CoP: these were binary questions on the presence of fatigue, problems with return to work or performance of daily activities and problems with memory and/or concentration. If any of these questions were answered with yes, OTs were consulted (Additional file 1: OT screening protocol). OT interventions addressed problems with fatigue and (insight in) physical capacity, (education on) cognitive functioning in daily activities and self-management. All REACH professionals were trained to regularly check for problems within other PICS domains but outside of the scope of their profession—such as psychological problems or worsening medical conditions—and informed general practitioners (GPs) when required.

PT started with functional exercises aimed at improving ability in ADL and gradually progressive resistance training to increase muscle strength. Interventions targeting exercise capacity progressed from functional, home-based training to in-practice aerobic training. Aerobic capacity was trained by first increasing the duration of the activity before increasing the intensity. If the participant's perceived exertion was > 4/10 on the Borg CR10 scale [37], therapists were to cease the exercise or the therapy session. The protocol identified 3 rehabilitation phases: (1) the (acute) home phase, (2) the (subacute) training phase at the PT clinic and (3) the evaluation (long-term follow up) phase. Progression between phases was left to the PTs professional judgment. Frequency of sessions averaged 2 half hour sessions per week in phase 1 and 30- to 60-min sessions twice a week in phase 2. In phase 3 participants often trained independently with irregularly scheduled supervised exercise sessions, as deemed necessary. The total duration of the REACH intervention was not specified a priori as decision-making depended on individual patient needs.

### Usual care group

The reference group consisted of participants receiving ‘usual care’, which was defined as ‘unrestricted clinical practice’, either sought through self-referral or recommended by the discharging hospital [38]. As no formal care pathway exists in the Netherlands for patients recovering from critical illness, we considered any participant who did not receive the REACH intervention, to be eligible for the usual care group.

Professionals involved in the usual care provision were not part of the REACH-network and did not receive additional training on (interventions targeting) PICS and application of the positive health concept. Some patients in the usual care group may not have received interventions from allied health professionals at all, dependent on their own preferences and the organization of health care.

### Outcomes

Primary (feasibility) outcomes were safety and optimal dose of the REACH program, patient and professional satisfaction, adherence to treatment and protocol, need for interdisciplinary referral and health care usage. Secondary outcomes were functional exercise capacity, self-perceived health status, health-related quality of life (HRQoL), return to work (RTW), prevalence of psychological problems (including symptoms of PTSD) and risk of undernutrition at time of hospital discharge.

Data collection took place between April 2019 and February 2021.

### Primary (feasibility) outcomes

Data on safety and optimal dose of the intervention were collected throughout the duration of the study by tracking adverse events and protocol deviations. Participant satisfaction with PT treatment was measured at 3- and at 6-month follow up with the Patient Reported Experience Measure (PREM, [39]). The PREM Physical Therapy is developed to measure patient experienced quality of the PT and the interventions received, estimating a global perceived effect and a net promotor score (NPS), which is calculated from the 0–10 score given to the question 'Would you recommend your PT to others with similar health problems?'. Scores to this question are grouped into ‘Promotors’ (score 9 or 10), ‘Passively satisfied’ (score 7–8) and ‘Detractors’ (score 0–6). The NPS is the derived result from the percentage promotors minus the percentage detractors.

Data on professional satisfaction and adherence to protocol were collected through a mixed-method approach using an online survey and a focus group session among REACH professionals, conducted at the end of the study. Information on referral need (DT and OT) was assessed as follows: DT need was assessed counting all cases with (risk of) undernutrition at time of hospital discharge and OT need was assessed at 3- and at 6-months after discharge by counting the cases applicable for OT based on the outcome of the screening protocol (Additional file 1). Data on health care usage were collected at 3- and 6-months after hospital discharge, using a self-reported questionnaire from a prior ICU follow-up study [40].

### Secondary outcomes

Physical measurements (conducted through home visits) and data collection of self-perceived health status, HRQoL and psychological status (GPS) were conducted at three timepoints: 1–2 weeks (T0), 3 months (T1) and 6 months (T2) after hospital discharge.

Functional exercise capacity was measured with the two-minute step test (TMST, [41]). The TMST is developed as part of the Senior Fitness Test and has shown to be a valid and reliable (ICC > 0.90) tool in older adults with and without morbidities, is practical in use in the home situation and can be safely conducted in frail (elderly) patients [42, 43]. Before testing, participants' vital signs were assessed by monitoring resting heart rate (RHR) blood pressure (BP) and oxygen saturation (SaO2) to determine safety and feasibility of test execution. Cut-off values for safe execution of the test were RHR ≤ 110, BP ≤ 180/100, and SaO2 > 90%. Other contra-indications for test execution were chronic heart failure, presence of chest pain, dizziness, wounds under the foot or inability to raise the knee to the height halfway between the iliac crest and the patella.

Self-perceived health status was assessed asking the participants to rate their health on a numeric rating scale (NRS) ranging from 0 (very bad) to 10 (excellent). Health-Related Quality of Life (HRQoL) was measured using the 36-item Short Form health survey (SF36, [44]). The SF36 consists of 8 subscales, which can be transformed into a physical component score (PCS) and mental component score (MCS) [45]. Return to work (RTW) data were collected via a self-reported questionnaire administered at 3- and 6 months [40].

The SNAQ65+ screening tool was used to determine undernutrition prevalence at hospital discharge (baseline). This tool categorizes nutrition status based on involuntary weight loss, upper arm circumference, appetite, and physical function in three categories: undernutrition (red), risk of undernutrition (orange) and no undernutrition (green) [35]. Prevalence of traumatic symptoms was determined at all 3 timepoints using the Global Psychotrauma Screen (GPS), a 22-item questionnaire designed to screen for a broad scope of potential trauma-related outcomes. The first 5 questions consist of the Primary Care PTSD Screen for DSM-5 (PC_PTSD-5, [46]), allowing for calculating an overall score for PTSD symptoms, where a score ≥ 3 indicates possible PTSD. A sum score of the remaining 17 questions provides a total score for GPS symptoms [47].

### Statistical analysis

Quantitative outcomes were analyzed descriptively and reported in raw counts, percentages, mean/SD or median/IQR, dependent on type and distribution of data. Due to the feasibility design of this study, no formal hypotheses testing on within and between group change over time were conducted—as the study was underpowered to test for effectiveness [32].

Baseline parameters between group were analyzed with the Mann–Whitney U test to explore if significant differences were present (α set at 0.05). For the secondary (clinical) outcomes, descriptive statistics at the 3 timepoints were calculated and converted to percentage of predicted values for outcomes where normative values exist. IBM SPSS version 27 was used. Qualitative data obtained through the focus group session were transcribed verbatim and combined with qualitative survey data. Further coding and thematic analysis of qualitative data took place and results are reported narratively.

### Ethical approval

As the REACH intervention is implemented in the form of quality improvement, the Medical Ethics committee of the Amsterdam University Medical Centers (location AMC) provided a waiver for the feasibility study (METC W18_237 # 18.282), but additional ethical approval was obtained for the physical measurements (2019_012, ABR NL 68475.018.19). Written informed consent was obtained from all participants in line with the Good Clinical Practice directives.

## Results

In total, 74 survivors of critical illness were referred for participation in the study, of which 16 were excluded because they were transferred to a long-term rehabilitation facility before home discharge, leaving 58 eligible participants. Application of the in- and exclusion criteria left a total of 43 participants, 19 participants were included in the intervention group and 24 in the usual care group.

**Fig. 1:**
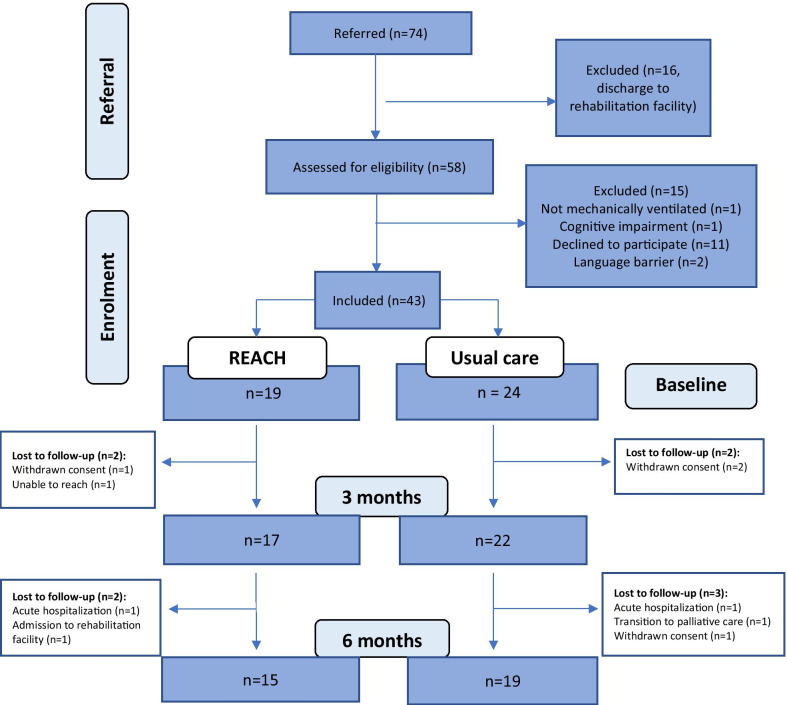
Flow of participants (consort diagram)

In each group 2 participants dropped out during the study due to an acute new medical event, unrelated to the intervention, requiring admission to hospital, rehabilitation- or palliative care facility. Loss-to-follow up occurred in both groups for the following reasons: withdrawn consent (REACH: *n* = 1, usual care: *n* = 3), unable to contact (REACH: *n* = 1). This resulted in a 6-month follow-up of 79.1% (*n* = 34). Due to the start of the COVID-19 pandemic halfway through this study, measurements were conducted telephonically during the 2-month complete lockdown in March/April 2020. Physical measurements continued as soon as protocols were put in place respecting social distancing and hygiene. This resulted in some missing data but no participant drop-out (Fig. 1).

### Baseline characteristics

Participant demographic and medical characteristics were similar between groups, except for age and hospital length of stay (LOS); participants in the intervention group were older than in the usual care group (median [IQR] age 63 [9] vs 54 [23], *p* 0.09) and had a significantly shorter median hospital LOS (23 vs 34 days, *p* 0.04). The majority of the participants were acutely admitted to ICU (REACH: 89.5%, usual care: 70.8%) and most had admission diagnoses of cardiorespiratory origin (Table 1).

**Table 1:** Population characteristics

Variable	REACH intervention group (*n* = 19)	Usual care group (*n* = 24)
Age (median/IQR)	63 (9)	54 (23)^a^
Gender, male (*n*, %)	14 (73.6)	15 (62.5)
ICU LOS (days) (median/IQR)	10 (16)	11 (12)^a^
MV duration (days) (median/IQR)	8 (10)	8.5 (12)^a^
Hospital LOS (days) (median/IQR)	23 (21)	34.5 (28)^b^
Admission diagnosis (*n*, %)		
Respiratory	12 (63.2)	11 (45.8)
Cardiac	2 (10.5)	7 (29.2)
Sepsis	3 (15.8)	2 (8.3)
Oncologic surgery	2 (10.5)	4 (16.7)
Admission category (*n*, %)		
Acute	17 (89.5)	17 (70.8)
Elective	2 (10.5)	7 (29.2)
SNAQ65+ screening score (*n*, %)	*n* = 19	*n* = 24
Red (undernutrition)	16 (84.2)	20 (83.3)
Orange (risk of undernutrition)	2 (10.5)	1 (4.2)
Green (no undernutrition)	1 (5.3)	3 (12.5)
Employment status (*n*, %)		
Employed*	7 (36.8)	10 (41.7)
Unemployed	12 (63.2)	14 (58.3)
Pensioner	4 (33.3)	5 (35.8)
Unemployed due to disability	4 (33.3)	3 (21.4)
Unemployed not due to disability	3 (25.0)	4 (28.6)
Family responsibilities	1 (8.4)	1 (7.1)
Student	–	1 (7.1)
Living situation (*n*, %)		
Living alone	6 (31.6)	6 (25.0)
Living with others^†^	13 (68.4)	18 (75.0)

*LOS* length of stay, *MV* mechanical ventilation, *SNAQ* short nutritional assessment questionnaire

^a^No significant between group differences (*p* > 0.05)

^b^Significant between group difference (*p* = 0.04)

^*^Includes permanent, casual, and self-employed employees

^†^Living in partnership, with child(ren), in student housing, living with friends or other family members

### Primary outcomes

#### Feasibility and safety of the intervention

No intervention related adverse events occurred and participants showed compliance to the treatment, as evaluated by the PTs providing the intervention: none of the patients included in the REACH group ceased treatment against the advice of the professional. REACH-PTs recognized that the treatment approach within the interdisciplinary network resulted in motivated patients showing high adherence to treatment, but identified challenges related to balancing care provision considering the patient's physical and mental capacity throughout the different stages of recovery. The frequency of PT treatment often had to be decreased when other disciplines were increasingly involved and/or demands from patients' environment intensified, to limit the strain on the patient.

#### Patient and professional satisfaction, adherence to treatment and protocol

Satisfaction scores were higher in the REACH group compared to the usual care group (NPS 92.8% vs 60.0% at 6 months). Evaluation of satisfaction among REACH professionals manifested the following positive feedback: applying the broader concept of health ('positive health') facilitated patient-centered care, in turn increasing patient satisfaction:

*I notice that [Positive health] is increasingly benefiting my way of communicating with patients [..] in which I have learned to place the patient first [..] and I really enjoy it. I notice patients are very satisfied ... with the treatment (REACH PT #3)*

Thematic analysis of the results of the focus group session with REACH-PTs (*n* = 11) revealed two themes: ‘Being part of the state-of-the-art’ and ‘Balancing patients’ needs with professional practice requirements’.

#### Being part of the state-of-the-art

The continuous professional development experienced by professionals within the interdisciplinary network, resulting from (online) meetings and training sessions, social media channels, discussion fora and monthly newsletters, increased awareness towards problems beyond the professional scope and led to changing one's daily practice:

*That meeting where we received information about nutrition and training opened my eyes! With every REACH patient, actually during my first consultation I check if the nutrition is in order, and always schedule a meeting with the dietitian in our center (REACH PT #6)*

Additionally, professionals experienced urgency in continuance of their professional development considering the complexity and heterogeneity of PICS, suggesting the network to be expanded with professionals from other disciplines, such as psychology and speech and language therapy (SLT). Similar emphasis was given to the need to expand the REACH network to a larger geographical area and ultimately to have  nationwide coverage. Being ready to provide fitting interventions for patients recovering from COVID-19 and being able to share knowledge and expertise to colleagues through national webinars was seen as a powerful opportunity:

*How great is it...no how terrible is it that we are in this COVID period, but how great is it that we can use the power of these webinars and online meetings. I really hope that we can take part in future research projects and continue meeting like this (FT#6)*

#### Balancing patients' needs with professional practice requirements

Thematic analysis revealed professional challenges regarding the delivery of optimal rehabilitation interventions for patients with PICS.

While the REACH CoP recommended usage of validated outcome measures such as the TMST, 2-min walk test (2-MWT) and 6-min walk test (6-MWT) for functional exercise capacity, this was deemed impractical by PTs providing the intervention, especially in patients with very low functional capacity or severe physical deconditioning. PTs identified the need for further validation of (functional) aerobic capacity tests for patients with PICS, such as cardio-pulmonary exercise testing (CPET) as soon as safely possible to establish training parameters and objectively evaluate (an increase in) exercise capacity.

Additionally, PTs identified limitations regarding financial compensation of PT sessions for patients with PICS. For patients for whom health insurance did not—or only limitedly—cover the expenses of the PT interventions, professionals often had to make difficult choices: to shorten the program or to provide sessions free of charge.

*You can design an intervention program with a desired frequency and for a desired duration but with limited coverage, you run out really quickly. Treatment is so dependent on individual circumstances and that makes it difficult. This patient I have, for example I have let him come for 2 additional months without letting him ... paying it myself because he has unemployment benefits only and I thought it important to get him back to how he was before (FT#7)*

Evaluating the application of the positive health concept, professionals indicated that the provided conversational tools were somewhat complicated and time-consuming in daily use, especially when met with patients with limited health literacy.

#### Health care usage and interdisciplinary referral need

The percentage of participants reporting hospital readmissions (acute and elective) was higher in the intervention group compared to the usual care group at both 3- and 6-month follow up (26.7% vs 9.5% at 3 months and 20.0% vs 6.7% at 6 months). The percentage of participants having planned hospital check-ups was initially similar between groups (3 months: REACH: 93.3% vs usual care: 95.2%) but decreased only in the REACH group at 6 months (REACH: 66.7% vs usual care: 93.3%). In the first 3 months, 212 PT sessions were received by 100% of the participants in the REACH group, versus 265 sessions to 76.2% of the participants in the usual care group. Between 3–6 months after hospital discharge the total PT sessions as well as the percentage of participants receiving PT had decreased (REACH: 152 sessions among 66.7% and usual care: 179 sessions among 73.3%).

A larger percentage of participants in the REACH group received OT compared to the usual care group (REACH 13.3% and 33.3% and usual care: 4.8% and 0% between 0–3 and 3–6 months respectively). The need to refer to OT seemed to increase over time (as the number of sessions and percentage of participants receiving OT increased in the REACH group between 3- and the 6-month follow up), while the percentage of participants needing DT interventions decreased somewhat over time (REACH: 53.3% and 40.0% and usual care: 47.6% and 33% between 0–3 and 3–6 months respectively). Visits of nursing practitioners were more frequent in the first 3 months after hospital discharge (REACH: 162 visits and usual care: 98 visits compared to the period between 3–6 months (REACH: 30 visits versus usual care: 27 visits). SLTs were not seen by anyone in the REACH group, and only 3 visits were reported by 1 participant in the usual care group in the period between 3–6 months.

**Fig. 2:**
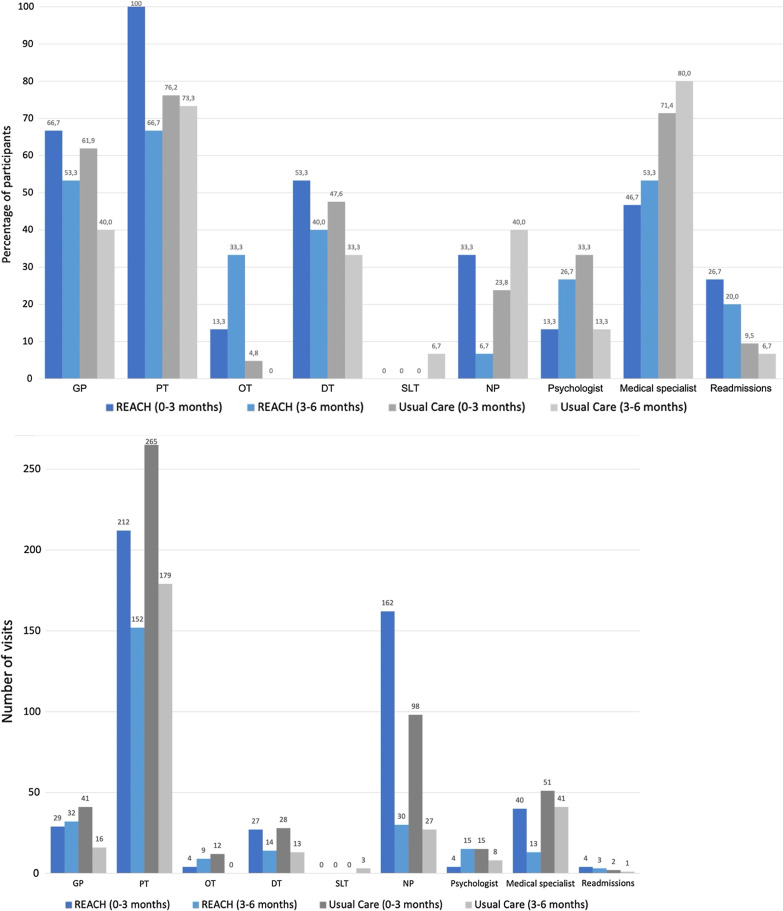
**a** Health care usage per group at 3 and at 6 months, expressed as percentage of participants. **b** Health care visits per group at 3 and at 6 months, expressed as total visits. *GP* general practitioner, *PT* physical therapist, *OT* occupational therapist, *DT* dietitian, *SLT* speech and language therapist, *NP* nursing practitioner

The frequency of consultations with medical specialists was lower in the REACH group compared to usual care, at both timepoints (REACH 46.7% [40 visits] and 53.3% [13 visits] vs usual care: 71.4% [51 visits] and 80.0% [41 visits] at 3- and 6-month follow up respectively). Appointments with psychologists occurred more often in the usual care group in the first 3 months (REACH: 13.3% [4 visits] vs usual care: 33.3% [15 visits]) which reversed between groups during the following 3 months (REACH: 26.7% [15 visits] and usual care: 13.3% [8 visits] (Fig. 2a, b).

### Secondary outcomes

#### Functional exercise capacity

Functional exercise capacity, measured with the TMST, was established in 72.1% of the participants directly after hospital discharge, in 86.5% at 3 months and in 93.8% at 6 months. Reasons for non-completion were unstable vital signs (elevated resting systolic or diastolic blood pressure or heart rate) or severe physical deconditioning, making the safe execution of the test impossible. Baseline performance was similar between groups (steps, mean [SD], REACH: 54 [18], usual care: 62 [33]). The greatest improvement in outcome was seen at 3 months follow up (steps, mean [SD], REACH: 82 [27] vs usual care: 94 [28]). At 6 months the improvement was still visible but tapered off (steps, mean [SD], REACH: 87 [31] vs usual care: 99 [28]). When comparing to normative values, mean steps improved to the lower limits of available norm values [48] in both groups, at 3- and at 6-month follow-up (Table 2).

**Table 2:** Secondary outcomes REACH versus usual care

Outcome	REACH			Control (usual care)		
	Discharge	3 months	6 months	Discharge	3 months	6 months
Functional exercise capacity (TMST)	*n* = 15	*n* = 16	*n* = 14	*n* = 16	*n* = 16	*n* = 16
Total steps (Mean/SD/∑)	54 ± 18, 809	82 ± 27, 1318	87 ± 31, 1213	62 ± 33, 992	94 ± 28, 1496	99 ± 28, 1590
Mean % norm (Mean/SD)						
Lower limit	0.65 ± 0.24	1.03 ± 0.33	1.05 ± 0.40	0.76 ± 0.39	1.15 ± 0.32	1.23 ± 0.32
Upper limit	0.48 ± 0.17	0.75 ± 0.24	0.77 ± 0.28	0.55 ± 0.29	0.84 ± 0.24	0.89 ± 0.24
NRS perceived health (0–10)	*n* = 19	*n* = 17	*n* = 13	*n* = 22	*n* = 19	*n* = 17
Median/IQR	5 (3)	7 (2)	8 (2)	6 (2)	7 (2)	8 (1)
HRQoL (SF-36) (Mean/SD)	*n* = 16	*n* = 15	*n* = 15	*n* = 22	*n* = 19	*n* = 15
PCS	34.1 ± 7.3	44.6 ± 11.1	43.9 ± 10.3	31.3 ± 9.5	40.7 ± 9.4	46.0 ± 7.3
MCS	42 ± 14.7	47.2 ± 10.2	51.0 ± 8.8	45.4 ± 11.7	52.9 ± 9.7	54.1 ± 7.0
Return to work						
Total prior employed	*n* = 7			*n* = 10		
Returned to work (*n*, %)		5 (71.4)	6 (85.7)		5 (50.0)	4 (40.0)
GPS	*n* = 16	*n* = 15	*n* = 15	*n* = 22	*n* = 20	*n* = 15
GPS Sum score (mean/SD)	5 ± 4	4 ± 3	3 ± 3	5 ± 4	3 ± 4	2 ± 2
Risk of PTSD (*n*, %)	4 (25.0)	1 (6.7)	1 (6.7)	4 (18.2)	3 (15.0)	1 (6.7)

TMST = Two-minute Step Test, ∑ = sum SD = Standard Deviation NRS = Numeric Rating Scale, IQR = Interquartile range, HRQoL = Health-related Quality of Life, SF-36 = Short Form 36 (Rand 36), PCS = Physical Component Score, MCS = Mental Component Score, SNAQ65+ = Short Nutritional Assessment Questionnaire 65+, GPS = Global Psychotrauma Screen, PTSD = Post Traumatic Stress Disorder

#### Self-perceived health status and health-related quality of life

Table 2 shows the outcomes at all three timepoints on the NRS perceived health. Data show a similar perceived improved health status between timepoints in both groups.

For HRQoL, baseline physical and mental component scores (PCS and MCS) for both groups are well below normative values and show a comparable recovery at 3- and 6 months, with minor differences observed between groups. Notably, neither group reaches normative values for PCS at 6 months [45].

#### Return to work

Of the participants who were employed prior to their ICU admission, 71.4% of the REACH participants and 50% of the usual care participants had returned to work (RTW) at 3 months. At 6 months 85.7% of the REACH participants reported RTW versus 40.0% in the usual care group. These data reflect both partial and complete RTW (Table 2).

#### Prevalence of undernutrition and psychotrauma

Results on the SNAQ65+ screening tool showed that 84.2% (*n* = 16) of the intervention group and 83.3% (*n* = 20) of the usual care group fell in the ‘undernutrition’ category (score ‘red’) at time of hospital discharge (Table 1). GPS results showed the presence of PTSD symptoms to be highest directly after hospital discharge (REACH: 25%, usual care: 18.2%) and decreasing with each following timepoint. GPS sum scores were the same for both groups at baseline and decreased over time (Table 2).

## Discussion

This study confirms the feasibility of the REACH program, an early individualized home-based rehabilitation intervention designed for patients with symptoms of Post-Intensive Care Syndrome (PICS). Our results show that collaboration within an interprofessional network consisting of hospital-based and primary care professionals, is a feasible method to provide rehabilitation interventions across the care continuum for survivors of critical illness. Early, home-based interventions were provided by expert professionals who were able to recognize patients’ needs across health domains. Commonly, hospital-based follow-up clinics are set up to identify aftercare needs for patients with PICS, but the timing of the first appointment is often delayed due to functional impairments patients might experience immediately after discharge [27]. As recommendations for rehabilitation interventions in the primary care setting are lacking [14], we believe our study might serve as an example for the implementation of healthcare interventions for patients with PICS-related symptoms across the care continuum, adding to the experience of a seamless transition from hospital to home.

Participants in the REACH group showed high motivation and adherence to treatment and reported higher satisfaction with PT treatment, when compared to the usual care group. This is contrary to findings of previous studies, which identified the heterogeneity of the population needing rehabilitation interventions after critical illness as a barrier for intervention adherence [22, 23, 25, 27, 49]. The extensive and long-term impairments of patients with PICS, potentially amplifying each other across health domains [50] could be explanatory for the fact that previous trials did not find significant differences in outcomes when compared to a control group. As trials need strict protocols and a 'one size fits all' design does not meet the needs of patients with PICS, different study designs and different types of interventions need to be explored. For this reason, the REACH intervention was characterized by a flexible, patient-centered, and tailored approach, founded in the principle of delivering the right care, at the right place, at the right time and by the right professional [51]. Providing the early interventions in the patients' homes could be another explanation for the low drop-out rate and high adherence to treatment in the REACH group, contrary to findings in studies with a larger, but similar population. Denehy et al. [23] investigated the effectiveness of an outpatient rehabilitation program for survivors of critical illness who were discharged home. Program completion rate was relatively low (41%), which was explained by sample heterogeneity, age, and comorbid disease [23]. Similarly, in a study by Berney et al. the post-ICU intervention was provided in the outpatient department of the hospital. Poor attendance and low adherence were explained by travel distance, poor social support and limited available time [22]. Our study shows that an individualized, home-based rehabilitation intervention increases patient adherence and satisfaction. Early home-based interventions are also likely to contribute to patient motivation and generally improve the transition from hospital to home [20, 27, 50].

A discussion point, however, is the identification of patients with (symptoms of) PICS at time of ICU- or hospital discharge. In this study we defined PICS as 'new or worsening symptoms in the physical, psychological or cognitive health domain, unrelated to the initial admission diagnosis or underlying conditions, at time of ICU- or hospital discharge'. A definition founded in the umbrella term postulated by the Society of Critical Care Medicine in 2012 [2] and applied in recent publications in absence of alternative diagnostic tools [33, 52, 53]. As no diagnostic tools for PICS exist at this moment [54], the population in our study cannot be formally identified as having PICS, although our secondary outcome data show that participants experienced impairments in physical, psychological, and/or cognitive domains. Clinical tools are needed to identify the presence of PICS and the extent of PICS-related disability, and although recently the development and validation of some tools have been investigated, further studies are urgently needed for better definition and understanding of PICS [54–58]. Working with the limitation of a not clearly defined population, we designed a patient-centered intervention embedded within an interdisciplinary collaborative network addressing the complex cluster of problems in patients with PICS conform recent recommendations [12, 33, 54].

Professionals within the REACH network showed great enthusiasm towards the opportunities for professional development, even on topics which were outside the scope of their discipline. Given a high prevalence of undernutrition at hospital discharge (> 80% in this study's population), PT interventions needed to be tuned with nutritional interventions. Our finding is in line with current literature, stating extreme loss of muscle mass in critically ill patients while reversal of the inflammatory, catabolic state takes time and effort [19, 59, 60]. Within the REACH interdisciplinary network, collaboration between PTs and DTs became a new standard of practice. Similar results were seen regarding the collaboration between PTs and OTs, but the amount of OT sessions received was limited, in both the REACH and usual care group. An explanation for this could be the early start of PT interventions, which in most cases combined with DT consults contributed to an already full rehabilitation schedule for patients. Balancing care provision while preventing to overload patients who are generally characterized by low physical and mental capacity, was a continuing challenge for professionals. Especially if, as recommended by REACH professionals, the interdisciplinary network is expanded with representatives from other disciplines such as psychologists and SLTs, the timing and intensity of the different consultations need to be reviewed considering individual rehabilitation goals.

**Fig. 3:**
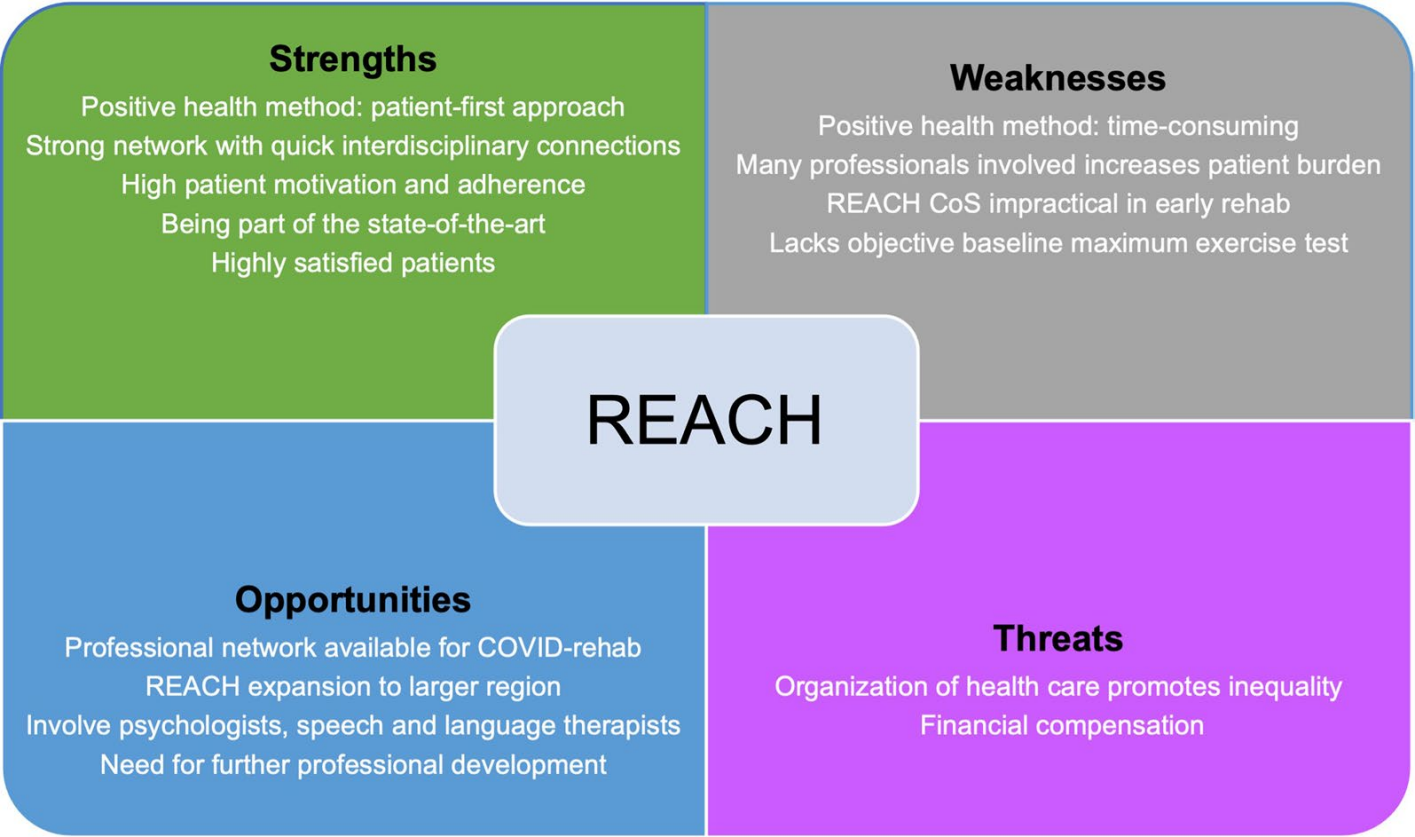
Strengths, weaknesses, opportunities and threats (SWOT) of the REACH program. CoS = Core Outcome Set

Additionally, recommendations were made for formalization of collaborative networks including representatives from medical insurance companies and general practitioners. Current organization of primary care PT in the Netherlands and the fact that no ICD-10 diagnostic code exists for PICS or PICS-related symptoms, were identified as barriers for the provision of state-of-the-art rehabilitation interventions as developed within this study (Fig. 3). Additionally, prior to the COVID-19 pandemic, PICS was largely unknown to or unrecognized by physicians responsible for referral of patients to rehabilitation professionals in the primary care setting [3, 61, 62]. An unfortunate result of this situation was that the REACH program could not be made available to everyone in need of rehabilitation after critical illness and hospital discharge. As our study shows, a larger number of visits to expensive medical specialist care (secondary or tertiary line of care) was reported within the usual care group when compared to REACH, while a smaller number of participants reported a higher total of PT sessions in the usual care group, which could be indicative of inefficiently organized healthcare. Current national initiatives towards guideline development and recommendations for recognition of PICS with an ICD-10 code will hopefully pave the way for efficient and equitable health care [54, 63, 64].

Collaborating within an interdisciplinary network to develop and provide a novel intervention for a population whose problems were largely unrecognized and inappropriately treated, facilitated REACH-professionals to become exemplars to colleagues within and outside of their own disciplines especially during the COVID-19 pandemic and the concurrent influx of patients with PICS-related symptoms.

Future studies should focus on further development of screening and assessment tools and intervention components for each of the disciplines involved in rehabilitation of patients with PICS. This should be done in co-creation, to ensure that all aspects of PICS can be addressed, and further professional development is encouraged.

### Strengths and limitations

Several limitations can be identified in our study.

First, there are some limitations to the recruitment and identification of the population in our study. Referral rate for the study is likely not representative of the true recruitment potential, as we had expected a larger study sample, based on available ICU- and hospital discharge data in the Netherlands. Therefore, it is likely that our study sample does not adequately represent the population in the ICUs of the 7 participating hospitals. Possible explanatory factors are of logistic nature, as eligible patients had to be identified within the ICU, while oral consent could only be obtained in the hospital wards. As one of our previous studies shows, the ward-stay is often experienced as turbulent by patients and family members, where the psychological effects of the ICU-stay start to sink in while hospital discharge is often organized swiftly [20]. We hypothesize that under these circumstances, recruitment for participation in research studies was difficult. Many patients declined to participate in research. Others did not see a need to continue PT at home, because they thought that they could recover without professional help or patients did not have health insurance covering PT interventions. Another explanation lies in the COVID-19 pandemic and its effect on workload and healthcare organization within the participating hospitals. In the academic hospitals, many scientific studies were initiated related to (recovery from) COVID-19. This likely decreased recruitment potential for our study.

Secondly, convenience sampling, fitting the feasibility design of this study, was applied. As a result of this, baseline differences were observed between groups, with regards to hospital length of stay (significantly shorter in intervention group) and age (a younger usual care group). This sampling method also likely contributed to bias in the reported results on satisfaction with and adherence to PT treatment, which therefore should be interpreted with caution. Additionally, we did not have access to data on pre-ICU functioning nor on severity of disease (APACHE II scores), and therefore important contextual information around our study population is lacking.

Thirdly, though the REACH program caters for 2 out of 3 pillars of the evidence-based practice paradigm (patient values and professional expertise), the scientific foundation is still lacking. The intervention provided did not follow a standardized protocol which might limit possibilities to draw inferences or be instructive towards the design of clinical trials. However, the heterogeneity of the population with PICS supports the need for exploration of different research designs to systematically evaluate patient-centered and individualized rehabilitation interventions. Also, our intervention was primarily focused on physical rehabilitation and while professionals were trained to observe impairments in the mental and cognitive health domains and referral structures were put in place, we did not succeed in addressing all components of PICS.

Lastly, self-reported questionnaires were used to obtain data on health care usage and return to work. These questionnaires, although used in earlier research, have not been validated and results should therefore be interpreted with caution. Data obtained do not allow us to perform a health economic evaluation comparing costs and outcomes of REACH with usual care, which would be essential to explore in future studies.

Strengths of this study lie in that we provided continuity of care for survivors of critical illness through the establishment of an interdisciplinary collaborative network. The REACH network shows potential for regional and national expansion and its right of existence was proven throughout the COVID-19 pandemic. The high satisfaction rates among the intervention participants indicate that individualized interventions with a patient-centered, holistic approach may be successful in the treatment of the heterogeneous population with PICS. Additionally, professionals in the network expressed feelings of achievement in their daily practice towards treatment of patients with PICS, resulting directly from interdisciplinary team discussions and continuous professional development sessions.

We obtained 6-month follow up data on 79.1% of our participants, despite the restrictive situation imposed to research studies during the COVID-19 pandemic, which provides us with moderate confidence towards our results.

## Conclusions

This study shows that it is safe and feasible to provide an early, home-based, rehabilitation intervention within the organization of an interdisciplinary professional network, for patients with symptoms related to PICS. High adherence to treatment and high satisfaction rates indicate that this treatment approach shows promise in addressing the complex needs of patients recovering from critical illness. Results show a potential impact on physical recovery and efficiency of health care organization, which can be used as a steppingstone towards further development of different components of interdisciplinary rehabilitation programs for patients with PICS, and as support for organization within interdisciplinary collaborative networks. Such networks can empower professionals to become professional experts and improve the quality of care provided to patients with PICS throughout the continuum. Future studies should be directed towards further development and effectiveness testing of different components of interdisciplinary rehabilitation programs, as well as health economic evaluations of care organized within such professional networks.

## Supplementary Information

**Additional file 1.** OT screening and referral protocol.

## Data Availability

The datasets generated and analyzed during the current study will be available from Figshare, DOI: 10.21943/auas.14806320.

## Abbreviations

2MWT: 2-Minute Walk Test
6MWT: 6-Minute Walk Test
ADL: Activities of Daily Living
APACHE: Acute Physiology and Chronic Health Evaluation
BP: Blood Pressure
CoP: Community of Practice
COVID-19: COrona VIrus Disease, 2019 novel coronavirus
CPET: CardioPulmonary Exercise Testing
DT: Dietary or Dietitian
FAC: Functional Ambulation Categories
GPS: Global Psychotrauma Screen
HRQoL: Health-Related Quality of Life
ICC: Intraclass Correlation Coefficient
ICD-10: International Statistical Classification of Diseases and Related Health Problems (10th revision)
ICU: Intensive Care Unit
ICU-PTs: Intensive Care Unit Physical Therapists
IQR: Intequartile Range
LOS: Length of Stay
MCS: Mental Component Scores
MV: Mechanical Ventilation
NPS: Net Promotor Score
NRS: Numeric Rating Scale
OT: Occupational Therapy or Occupational Therapist
PC-PTSD-5: Primary Care PTSD Screen for DSM-5
PCS: Physical Component Scores
PICS: Post-Intensive Care Syndrome
PREM: Patient Reported Experience Measures
PT: Physical Therapy or Physical Therapist
PTSD: Post-Traumatic Stress Disorder
REACH: REhabilitation After Critical illness and Hospital discharge
REACH-PT: Physical Therapist within the REACH network
RHR: Resting Heart Rate
RTW: Return to Work
SaO2: Oxygen Saturation
SD: Standard Deviation
SF36: Short Form 36
SLT: Speech and Language Therapist
SNAQ65+: Short Nutritional Assessment Questionnaire (for adults 65+)
TMST: Two-Minute Step Test
WHO: World Health Organization
