# Supplementary material for: Feasibility of a home-based interdisciplinary rehabilitation program for patients with Post-Intensive Care Syndrome: the REACH study
Source: Crit Care. 2021 Aug 5;25:279. doi: 10.1186/s13054-021-03709-z (PMC8339801; doi:10.1186/s13054-021-03709-z)
Supplement: Supplementary file 1 — Additional file 1. OT screening and referral protocol. [file 13054_2021_3709_MOESM1_ESM.pdf]

Additional file: Occupational Therapy Screening Questions and referral protocol

with: Major et al. ***Feasibility of a home-based interdisciplinary rehabilitation program for patients with Post-Intensive Care Syndrome: the REACH study***

1. Do you experience difficulty with resuming work activities?

☐ *No*

☐ *Yes --> refer to OT*

2. Do you experience difficulty with limited energy or fatigue? Or do you feel that fatigue has a negative effect on your daily life?

☐ *No*

☐ *Yes --> refer to OT*

3. Do you experience difficulty with performing your daily activities?

☐ *No*

☐ *Yes--> refer to OT*

4. Do you experience difficulty with your memory and/or concentration?

☐ *No*

☐ *Yes--> refer to OT*

In case the answer to any of these questions is 'Yes' discuss referral to OT with the participant. If the participant agrees to referral, contact the nearest REACH-OT.
